# Association of C-Reactive Protein, Interleukin-1 Receptor Antagonist and Adiponectin with the Metabolic Syndrome

**DOI:** 10.1155/2007/93573

**Published:** 2007-11-25

**Authors:** Juha Saltevo, Mauno Vanhala, Hannu Kautiainen, Esko Kumpusalo, Markku Laakso

**Affiliations:** ^1^Department of Internal Medicine, Central Hospital of Middle Finland, 40620 Jyväskylä, Finland; ^2^Department of General Medicine, Central Hospital of Middle Finland, 40620 Jyväskylä, Finland; ^3^Laukaa Health Center, 41341 Laukaa, Finland; ^4^Medcare Foundation, 44100 Äänekoski, Finland; ^5^Department of Public Health and Clinical Nutrition, University of Kuopio, 70211 Kuopio, Finland; ^6^Unit of General Practice, Kuopio University Hospital, 70211 Kuopio, Finland; ^7^Department of Medicine, University of Kuopio, 70211 Kuopio, Finland

## Abstract

This Finnish population-based study, mean age 46 years, evaluates the association of high-sensitivity C-reactive protein (hs-CRP), interleukin-1 receptor antagonist (IL-1Ra), and adiponectin with the NCEP and IDF definitions of metabolic syndrome (MetS). Adiponectin levels were higher, hs-CRP and IL-1Ra levels lower in subjects without MetS compared to subjects with MetS. If MetS was present according to both IDF and NCEP criteria, BMI, waist, triglycerides, hs-CRP, and IL-1Ra were significantly higher compared to subjects who had MetS according to either only IDF or only NCEP criteria. The hs-CRP, IL-1Ra, and adiponectin linearly correlated with the number of the components of MetS according to both definitions. Decreased levels of adiponectin and increased levels of hs-CRP and IL-1Ra are tightly associated with the components of MetS. Individuals who had MetS according to both criteria had the most adverse changes in cardiovascular risk factors.

## 1. INTRODUCTION

Metabolic syndrome (MetS) means a clustering of multiple cardiovascular risk factors in an individual. Several definitions of this syndrome have been presented. The first definition was given by the World Health Organization (WHO) in 1999. The primary aim of this definition was to identify individuals with high risk for cardiovascular disease (CVD) [1].

The National Cholesterol Education Program (NCEP) Adult Treatment Panel III introduced a new definition for the MetS in 2001 [2]. This definition was modified in 2004 when the cutoff point for the fasting glucose level was lowered from ≥6.1 mmol/L to ≥5.6 mmol/L based on the new definition of impaired fasting glucose of the American Diabetes Association [3]. The purpose of the NCEP definition was to identify people who are at high long-term risk for CVD and/or type-2 diabetes. The most recent definition has been presented by the International Diabetes Foundation (IDF) in 2005 [4, 5]. It is a modification from the NCEP definition, but in contrast to the NCEP definition, abdominal obesity (large waist) is mandatory for the MetS in the IDF definition, because it was considered to be the most important etiological component of the syndrome.

Adipose tissue has also shown to be an active endocrine organ secreting many hormones and mediators regulating glucose metabolism and the risk of CVD. One of these hormones is adiponectin that has been found to be decreased in individuals with obesity, the MetS, and type-2 diabetes [6, 7]. Adiponectin does not modulate only glucose and lipid metabolism, but also the immune system. Thus, adiponectin is currently recognized as an anti-inflammatory adipocytokine [8].

Obesity is an inflammatory condition which may lead to chronic activation of the innate immune system, which in turn could cause a progressive impairment of glucose tolerance and lead to diabetes or CVD [9, 10]. In agreement with this hypothesis increased levels of high-sensitivity C-reactive protein (hs-CRP), an acute-phase protein, has shown to predict coronary heart disease (CHD) [11–13] and the development of the MetS [14], type-2 diabetes [15], and the development of both CVD and type-2 diabetes [16, 17]. In a 12-year follow-up study including elderly women, an increment of 1 pg/ml of baseline hs-CRP level was associated with a 37% increase in the risk of the MetS defined by the NCEP criteria [18]. Based on these studies, several investigators have suggested that high hs-CRP should be added in the definition of the MetS because it connects proinflammation, central adiposity, and MetS, and could be measured in clinical practice [19, 20].

Interleukin-1 receptor antagonist (IL-1Ra), a naturally occurring antagonist of the proinflammatory cytokines IL-1α and IL-1β, reflects inflammatory response. It is a natural compensatory mechanism for IL-1-induced disease process [21], and it is elevated in subjects with the MetS [22]. IL-1Ra has anti-inflammatory properties, because it competitively binds to membrane receptors of IL-1β. It is also an acute phase reactant produced by liver during the inflammatory state [23]. Thus, low circulating concentrations of adiponectin and high levels of hs-CRP and IL-1Ra are markers of a proinflammatory state.

No previous studies have been published on the role of hs-CRP, IL-1Ra, and adiponectin in relation to different definitions of the MetS in the same population. The aim of the present study was to evaluate the inflammatory differences and associations between the inflammatory markers and adiponectin with the MetS according to the NCEP and the IDF definitions.

## 2. MATERIALS AND METHODS

### 2.1. Subjects

The study population consisted of middle-aged Caucasian subjects (N=1294) born in 1942, 1947, 1952, 1957, and 1962 (the entire age group) in Pieksämäki, eastern Finland. All participants gave an informed written consent. The study protocol was approved by ethics comittee of the Kuopio University Hospital and the University of Kuopio.

Altogether 923 of 1294 subjects (71.3%) participated in this cross-sectional study in 1997–1998. All participants filled a standard questionnaire including questions about medication, smoking habits, and physical activity. We excluded from statistical analysis 18 subjects (7 men and 11 women) with hs-CRP concentrations ≥10.0 mg/L to exclude possible cases of acute infections and other occult diseases. Additional three women were lost from statistical analysis because of missing data for the components of the MetS.

### 2.2. Definitions of the metabolic syndrome

MetS was defined based on the criteria proposed by NCEP [3] and IDF [4, 5]. According to the NCEP criteria, the MetS was defined as the presence of 3 or more of the following cardiovascular risk factors: abdominal obesity (waist circumference >102 cm in men and >88 cm in women), triglycerides ≥1.7 mmol/L(150 mg/dL) or drug treatment for dyslipidemia, HDL cholesterol <1.03 mmol/L (40 mg/dL) in men and <1.29 mmol/L (50 mg/⁢dL) in women or drug treatment for dyslipidemia, blood pressure ≥130/≥85 mm Hg or drug treatment for hypertension, and fasting plasma glucose ≥5.6 mmol/L (100 mg/⁢dL) or treatment for diabetes.

According to the IDF criteria, the MetS was defined to be present in individuals who had central obesity (waist circumference ≥94 cm in men and ≥80 cm in women) plus any 2 of the following factors: triglycerides >1.7 mmol/L or drug treatment for dyslipidemia, HDL cholesterol <1.03 mmol/L in men and <1.29 mmol/L in women or drug treatment for dyslipidemia, blood pressure ≥130/≥85 mm Hg or drug treatment of previously diagnosed hypertension, and fasting plasma glucose ≥5.6 mmol/L or previously diagnosed and treated type-2 diabetes.

### 2.3. Clinical and laboratory methods

Height and weight were measured to the nearest 0.5 cm and 0.1 kg, respectively. BMI was calculated as weight (kg) divided by height (m) squared. The waist was measured at the midpoint between the lateral iliac crest and the lowest rib to the nearest 1 cm. Trained nurses measured blood pressure (BP) twice in subjects at sitting position after a 15-minute rest with a mercury sphygmomanometer. The latter value was used in statistical analysis.

Fasting blood samples were drawn after 12 hours of fasting. Plasma was separated by centrifugation for the determination of fasting insulin and the samples were frozen immediately. Insulin was determined by Phadeseph insulin radioimmunoassay (RIA) 100 methods (Pharmacia Diagnostics AB, Uppsala, Sweden). Glucose concentration was measured by automated colorimetric method (Peridochrom glucose GOD-PAP, Boehringer, Germany). Serum cholesterol and triglycerides were measured from fresh serum samples with enzymatic colorimeter methods (CHOD-PAP, GPO-PAP, Boehringer Mannheim GmbH, Germany). Serum HDL cholesterol was measured using the same methods after the precipitation of low-density cholesterol and very low-density lipoprotein cholesterol by phosphotungstic acid and magnesium.

Serum adiponectin was determined with an enzyme immunoassay (human adiponectin ELISA Kit, B-Bridge International Inc, Calif, USA). Plasma concentration of IL-1Ra was measured with high-sensitivity assay kits from R&D Systems. hs-CRP was measured with an Immulite analyzer and a DPC high-sensitivity CRP assay (hs-CRP). Adiponectin, hs-CRP, and IL-1Ra were measured in 2002 at the same time. Before this measurement, the samples werefrozen andstored at −70°C.

In this study, we used the quantitative insulin sensitivity check index (QUICKI) as a marker of insulin sensitivity. It is an alternative method to measure insulin sensitivity in large population studies and was calculated as follows: QUICKI = 1/(log FPI + log FBG), where FPI = fasting plasma insulin level expressed as mU/l, and FBG = fasting plasma glucose level expressed as mg/dL [24].

### 2.4. Statistical analysis

The results are expressed as mean ± standard deviation (SD). Confidence intervals for the means were obtained by bias-corrected and accelerated bootstrapping (5000 replications). Statistical comparison between groups was performed by t-test or analysis of variance (ANOVA) with bootstrap-type tests, when appropriate. Bootstrap-based multiplicity adjustment will be applied to correct levels of significance for multiple testing when appropriate [25]. The agreement between the definitions was determined by the kappa statistic (κ). The level of agreement is considered poor if κ<0.20, fair if κ=0.21–0.40, moderate if κ=0.41–0.60, substantial if κ=0.61–0.80, and very good if κ>0.80 [26].

## 3. RESULTS

Altogether 923 of 1294 invited subjects (71.3%) participated in this study. Table 1 presents demographic, clinical, and biochemical characteristics of study subjects. Waist, blood pressure, triglyceride, and glucose levels were higher and HDL cholesterol concentrations lower in men than in women (P<.001). Median adiponectin concentration was lower in men than in women (4.9 versus 7.9 μg/ml, P<.001), whereas median hs-CRP was higher in women than in men (1.5 versus 1.3 pg/ml, P=.035).

Among the 923 participants, the prevalence of the MetS according to the IDF definition was 38% in men and 34% in women. According to the NCEP criteria, the corresponding numbers were 34% in men, and 27% in women. In women, the agreement (κ) between the IDF and NCEP criteria was 0.75 (95% Cl 0.68 to 0.81) and in men 0.60 (95% Cl 0.52 to 0.68).

Table 2 shows that adiponectin levels were significantly (P<.001) lower in males and females in subjects with the Mets compared to subjects without Mets, independently of the definition used. On the other hand, the levels of hs-CRP and IL-1Ra were significantly higher in subjects with the MetS compared to subjects without the MetS in both genders.

Insulin sensitivity index measured by QUICKI was significantly higher in subjects without the MetS than with MetS according to both definitions without any gender difference (IDF criteria: 0.35±0.18 versus 0.32±0.02, P<.001; NCEP criteria: 0.35±0.02 versus 0.32±0.02, P<.001).

Table 3 shows that when the MetS was present according to bothIDF and NCEP definitions, waist and triglyceride levels were significantly higher and HDL cholesterol lower compared to those who had the MetS just according to the IDF, but not NCEP criteria, or MetS just according to the NCEP criteria, but not IDF-criteria in both genders.

Table 4 shows that the mean hs-CRP level was 1.00 pg/ml in men having the MetS according only to the NCEP definition, 1.52 pg/ml having the MetS according only to the IDF definition, and 1.73 pg/ml when both definitions (IDF and NCEP) simultaneously were present (P=.033 between groups and linearity 0.020). The mean hs-CRP level in women was 0.81, 1.45, and 2.62 pg/ml (P<.001 between the groups, linearity 0.010), respectively. The corresponding levels of adiponectin and IL-1Ra are also shown in Table 4.

**Figure 1:**
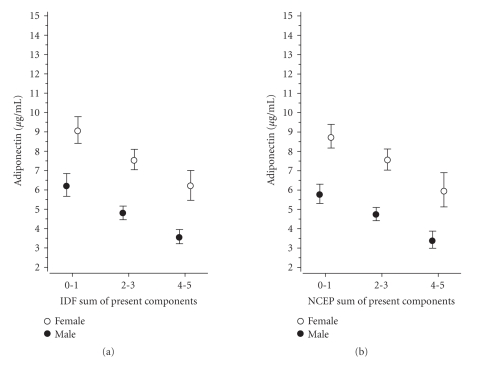
Relationship between adiponectin and the number of the components of the metabolic syndrome according to the IDF and NCEP criteria (95% confidence intervals obtained by bias-corrected and accelerated bootstrapping (5000 replications)).

**Figure 2:**
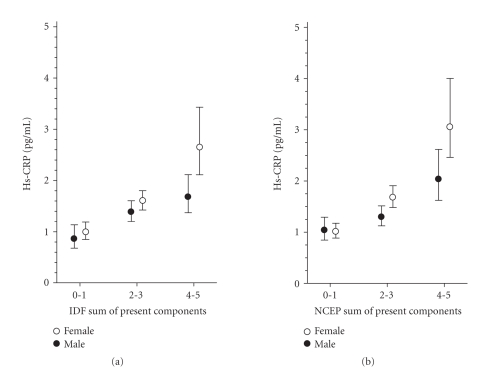
Relationship between high-sensitivity C-reactive protein (hs-CRP) and the number of the components of the metabolic syndrome according to the IDF and NCEP criteria (95% confidence interval obtained by bias-corrected and accelerated bootstrapping (5000 replications)).

**Figure 3:**
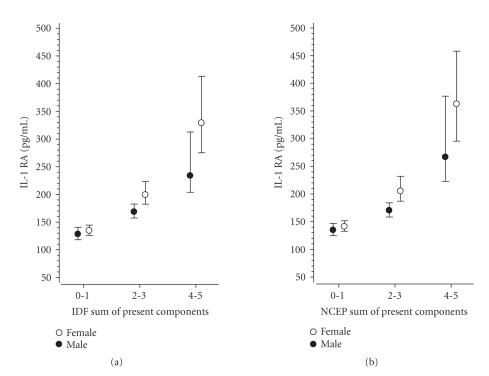
Relationship between interleukin-1 receptor antagonist (IL-1Ra) and the number of components of the metabolic syndrome according to the IDF and NCEP criteria (95% confidence interval obtained by bias-corrected and accelerated bootstrapping (5000 replications)).

Figures 1–3 show the correlation of adiponectin, hs-CRP, and IL-1Ra with the number of the components of the MetS present (0-1, 2-3, 4-5) according to the IDF and theNCEP criteria in both genders (P for linearity <.001 in all definitions).

## 4. DISCUSSION

Our study shows that proinflammatory cytokines and adiponectin are likely to be central components of the MetS. Cytokine levels were higher and adiponectin levels lower in subjects who had the MetS according to both definitions (IDF, NCEP) compared to subjects who had the MetS according to only one definition. Furthermore, the only-IDF definition group of the MetS had higher levels of hs-CRP and IL-1Ra compared to the group with only-NCEP definition, probably due to abdominal obesity as the central criterion for the syndrome. The same stronger relationship with the IDF definition compared to the NCEP was found in Chines population with hs-CRP [27]. Subjects with the MetS defined according to only NCEP criteria had quite normal levels of adiponectin and inflammatory markers, which may indicate that these subjects are not at so high risk for CVD and/or diabetes. On the other hand, the levels of triglycerides were higher and HDL cholesterol tended to be lower in this only NCEP-population. In a recent study, quite similar risk for CVD and diabetes were observed in subjects having the MetS according to the IDF, NCEP, and WHO definitions [28].

Excess adipose tissue can contribute to inflammation in several ways. First, ectopic fat storage induces lipotoxicity promoting intracellular inflammatory response or altered adipokine production [29]. Second, hypoadiponectinemia may result by interactions of genetic factors in the adiponectin gene itself and environmental factors causing obesity, which leads to insulin resistance and MetS [8]. In the ADOPT study including recently diagnosed, drug naïve type-2 diabetic subjects inflammatory markers were strongly related to the numberof the components of the MetS [30]. Anyhow, in several other studies, different measures of insulin resistance and cardiometabolic risk factors have correlated significantly with intra-abdominal adiposity [31–33].

Our study confirmed that levels of adiponectin, hs-CRP, and IL-1Ra were similarly and linearly correlated with the number of components of the MetS according to the IDF and NCEP definitions in both genders. Similar results have been reported previously with respect to levels of hs-CRP [30, 34, 35] and adiponectin [36–39] in different populations. In the Finnish Diabetes Prevention Study, hs-CRP was the best immunological predictor for the progression from impaired glucose tolerance to overt type-2 diabetes [40]. IL-1Ra has been shown to be the most sensitive marker of cytokine response in the prediabetic state among the offspring of type-2 diabetic patients, probable protecting human beta-cells from glucose-induced functional impairment [41], and the levels of IL-1Ra are decreased when type-2 diabetes develops [42]. However, longitudinal studies are missing to indicate whether or not IL-1Ra levels predict the development of diabetes or CVD. Our findings show that according to any definition of the MetS, conventional cardiovascular risk factors, insulin resistance measured by QUICKI, low adiponectin, and high-level proinflammatory markers cluster in the same individuals.

The prevalence of the MetS was 38% in men and 34% in women according to the IDF definition, and 34% and 27%, respectively, according to the NCEP definition. Our results agree with previous results from Finland [43]. The prevalence of MetS defined by IDF was somewhat higher, especially in women. On the other hand, the level of agreement between both definitions was better in women. Mostly both definitions of the MetS identified the same high-risk individuals, but the IDF definition identified more reliably individuals with more unfavourable proinflammatory parameters than did the NCEP definition. Subjects having the MetS according to both IDF and NCEP criteria had significantly higher BMI, waist circumference, triglycerides and inflammatory markers and lower levels of adiponectin and HDL cholesterol compared to subjects having the MetS according to only either IDF or NCEP criteria.

The strength of our study is that it includes a substantial number of subjects selected from 5 different age groups without any exclusion criteria in the same town. Therefore, we could reliably compare the correlations of IL-1Ra with hs-CRP and adiponectin in the same study population, which is fairly homogeneous regarding age, BMI, and origin. We could demonstrate that IL-Ra is correlated with Mets similarly as hs-CRP and adiponectin. The limitation of our study is that we measured total adiponectin, but not the high molecular weight (HMW) multimer of adiponectin, which has been shown to be a better marker for the MetS than is the total adiponectin level [44, 45].

## 5. CONCLUSIONS

We conclude that decreased levels of adiponectin and increased levels of hs-CRP and IL-1Ra possibly reflect the same phenomenon, and correlate linearly with the number of the components of the MetS according to the IDF and NCEP definitions. The levels of inflammatory markers (hs-CRP and IL-1Ra) are higher among patients with MetS defined only by the IDF definition compared to the only NCEP defined ones. More longitudinal follow-up studies are needed to investigate whether or not these new markers of the MetS increase the predictive power with respect to future risk of type-2 diabetes and CVD in different populations.

## Figures and Tables

**Table 1:** Demographic, clinical, and biochemical characteristics of the study population.

Variables	Men (N=405)	Women (N=497)	P-value
	Mean (SD)	Mean (SD)	
Demographic			
Age, years	46 (6)	46 (6)	
Body mass index, kg/m²	26.7 (3.8)	26.3 (4.9)	.15
Waist, cm	93.8 (10.6)	83.3 (12.2)	<.001

Clinical			
Blood pressure, mmHg			
Systolic	137 (17)	131 (17)	<.001
Diastolic	84 (10)	79 (9)	<.001

Biochemical			
HDL cholesterol, mmol/L	1.3 (0.3)	1.5 (0.3)	<.001
Triglycerides, mmol/L	1.7 (1.3)	1.2 (0.6)	<.001
FP-glucose, mmol/L	5.9 (0.6)	5.6 (0.5)	<.001
FP-insulin, mU/L	10.7 (5.9)	9.8 (6.5)	.033
hs-CRP, pg/ml	1.3 (1.5)	1.5 (1.7)	.035
IL-1Ra, pg/ml	172 (131)	192 (167)	.16
Adiponectin, μg/ml	4.9 (2.7)	7.9 (4.4)	<.001

HDL = high-density lipoprotein, hs-CRP = high-sensitivity C-reactive protein, IL-1Ra = interleukin-1 receptor antagonist, SD = standard deviation.

**Table 2:** Adiponectin, interleukin-1 receptor antagonist (IL-1Ra), and high-sensitivity C-reactive protein (hs-CRP) levels according to the IDF and the NCEP definitions of metabolic syndrome in men and women.

		Definitions of metabolic syndrome					
		IDF			NCEP		
	Not present	Present	P-value^†^	Not present	Present	P-value^†^
	Mean (SD)	Mean (SD)	Mean (SD)	Mean (SD)		

Men						
No of subjects, (%)	*250 (62)*	*155 (38)*		*268 (66)*	*137 (34)*
Adiponectin, *μ*g/ml	5.31 (2.75)	4.15 (2.46)	<.001	5.29 (2.83)	4.04 (2.23)	<.001
IL-1Ra, pg/ml	145 (72)	217 (182)	<.001	153 (84)	210 (187)	<.001
hs-CRP, pg/ml	1.10 (1.39)	1.67 (1.62)	<.001	1.18 (1.44)	1.57 (1.61)	0.04

Women						
Number	*328 (66)*	*169 (34)*		*362 (73)*	*135 (27)*
Adiponectin, μg/ml	8.44 (4.69)	6.89 (3.72)	<.001	8.34 (4.63)	6.77 (3.67)	<.001
IL-1Ra, pg/ml	145 (77)	284 (242)	<.001	154 (106)	294 (243)	<.001
hs-CRP, pg/ml	1.10 (1.26)	2.32 (2.09)	<.001	1.15 (1.26)	2.49 (2.24)	<.001

^†^Bootstrap type t-test with bootstrap-based multiplicity adjustments (5000 replications). 95% confidence interval obtained by bias-corrected and accelerated bootstrapping (5000 replications).

**Table 3:** Demographic, clinical, and biochemical characteristics of subjects with the metabolic syndrome.

Variables	Definitions of the metabolic syndrome						P-value between	
	Only NCEP		Only IDF		Both IDF and NCEP			
	Male	Female	Male	Female	Male	Female	Males	Females
	(N=28)	(N=10)	(N=46)	(N=44)	(N=109)	(N=125)		
	Mean (SD)	Mean (SD)	Mean (SD)	Mean (SD)	Mean (SD)	Mean (SD)		
Demographic								
Age, years	48 (7)	49 (5)	48 (6)	47 (6)	47 (6)	48 (6)	0.87	0.76
Body mass index, kg/m^2^	25.7 (1.9)	24.1 (1.9)	27.1 (1.7)	27.2 (2.3)	30.8 (3.4)	31.5 (5.1)	<.001	<.001
Waist, cm	89 (4)	76 (4)	97 (2)	85 (2)	106 (9)	97 (11)	<.001	<.001

Clinical								
Blood pressure, mmHg								
Systolic	141 (12)	134 (6)	142 (19)	134 (16)	144 (16)	141 (16)	.61	.035
Diastolic	87 (11)	80 (10)	87 (9)	82 (7)	89 (9)	85 (7)	.32	.038

Biochemical								
HDL cholesterol, mmol/L	1.2 (0.3)	1.2 (0.2)	1.4 (0.3)	1.5 (0.4)	1.1 (0.2)	1.3 (0.3)	<.001	.001
Triglycerides, mmol/L	2.5 (1.5)	1.6 (0.6)	1.6 (0.9)	1.3 (0.6)	2.4 (1.8)	1.7 (0.8)	.007	.004
FP-glucose, mmol/L	6.1 (0.6)	5.8 (0.4)	6.1 (0.5)	5.9 (0.6)	6.3 (0.7)	6.0 (0.6)	.061	.23

HDL = high-density lipoprotein.

**Table 4:** Levels of adiponectin, interleukin-1 receptor antagonist (IL-1Ra), and high-sensitivity C-reactive protein (hs-CRP) according to the IDF and the NCEP definitions of the metabolic syndrome, according to gender.

Variables	Definitions of metabolic syndrome			P-value^†^	
	Only NCEP	Only IDF	Both IDF and NCEP	Between group	Linearity
Men					
Number	28	46	109		
Adiponectin, *μ*g/ml, mean (SD)	4.91 (2.54)	4.94 (3.05)	3.81 (2.10)	.021	.034
IL-1Ra, pg/ml, mean (SD)	140 (73)	189 (118)	228 (203)	.032	.018
Hs-CRP, pg/ml, mean (SD)	1.00 (0.98)	1.52 (1.40)	1.73 (1.70)	.033	.020

Women					
Number	10	44	125		
Adiponectin, *μ*g/ml, mean (SD)	8.48 (5.08)	7.62 (4.18)	6.63 (3.52)	.25	.14
IL-1Ra, pg/ml, mean (SD)	146 (84)	222 (216)	305 (248)	.004	.044
hs-CRP, pg/ml, mean (SD)	0.81 (1.06)	1.45 (1.18)	2.62 (2.26)	<.001	.010

^†^Bootstrap-type ANOVA.
